# Variability in individual musculoskeletal response is not increased by countermeasures during bed rest

**DOI:** 10.3389/fphys.2025.1645482

**Published:** 2025-10-14

**Authors:** Jonas Böcker, Marie-Therese Schmitz, Leona Hoffmann, Wilhelm Bloch, Jörn Rittweger

**Affiliations:** ^1^ Department of Muscle and Bone Metabolism, Institute of Aerospace Medicine, German Aerospace Center, Cologne, Germany; ^2^ Institute of Medical Biometry, Informatics and Epidemiology (IMBIE), University Hospital Bonn, Bonn, Germany; ^3^ Institute of Cardiovascular Research, Molecular and Cellular Sport Medicine, German Sport University, Cologne, Germany; ^4^ Department of Pediatrics and Adolescent Medicine, University Hospital of Cologne, Cologne, Germany

**Keywords:** between-subject variation, variability quantification, countermeasure variability, inter-individual adaptations, muscle atrophy, bone loss

## Abstract

**Introduction:**

Under weightlessness conditions, there is a loss of bone and muscle mass. However, these adaptations are subject to great inter-individual variability. As an analogue to weightlessness, bed rest studies are carried out on Earth, which also serve to test countermeasures counteracting bone and muscle loss. Since study participants react differently to any interventions such as countermeasures, it can be assumed from a statistical point of view that the between-subject variation (BSV) is inflated when combining bed rest with countermeasure exercises. The aim of this study was therefore to examine whether a countermeasure has an effect on the magnitude of BSV.

**Methods:**

To this purpose, the decrease in muscle cross-sectional area and bone mineral content was analyzed at different measurement sites of 123 subjects in the control and intervention groups from six bed rest studies that tested different countermeasures. A novel statistical approach was chosen enabling quantification of the specific amount of variability after countermeasures (*U*_*CM*_).

**Results:**

The comparison of the observed variability (*U*_*Obs*_) between intervention and control groups showed no difference (all p ≥ 0.08), suggesting that BSV was not affected by the countermeasures. *U*_*CM*_ was negligible in the context of the overall variability, indicating that it plays a subordinate role in whether a crew member responds weakly or strongly to a training intervention.

**Conclusion:**

But rather, the extent to which, the astronaut reacts to unloading is the main factor influencing variability.

## 1 Introduction

The human body adapts to environmental changes in several manners. Thus, the musculoskeletal system reacts with adaptations to a lack of mechanical loading which occurs during microgravity (Fitts et al., 2010; LeBlanc et al., 2007; Rittweger et al., 2018; Vico et al., 2000). These adaptations include muscle wasting and bone loss in the lower extremities (Man et al., 2022; Stavnichuk et al., 2020) as these tissues are highly responsive (Juhl et al., 2021). These losses can be up to 24% after 6 months of microgravity for the muscle (LeBlanc et al., 2000) and averaged 1–1.5% bone mineral content (BMC) loss per month (Pavy-Le Traon et al., 2007; Juhl et al., 2021; LeBlanc et al., 2000; Vico et al., 2017). It has been shown that there is individual variability in response to spaceflight (Sibonga et al., 2015; Scott et al., 2023), but due to the different conditions and the progression of bone loss during the missions, this variability has to be interpreted with care (Scott et al., 2021; Gabel et al., 2021; Vico et al., 2017). To reveal the sources of these adaptations, bed rest studies are performed as these are highly standardized Earth environments designed to be analogues to microgravity. As previously published in Figure 3 in Böcker et al. (2022), the adaptations after bed rest are subject to great between-subject variation (BSV), which could be explained by differences in anthropometrics, genetics, and daily activities prior to bed rest. BSV is an aspect that should be given great attention, because future long-term missions should not only focus on the average loss of bone and muscle in crew members, but in particular on those crew members who show the greatest risk of losing bone and muscle mass. Thus, inter-individual variability could play an important role in future crew selection (Scott et al., 2021).

Of course, another aspect influencing the variability is the fact that humans react differently to any intervention, especially training interventions. These adaptations can have a wide range from being a great responder to being a non-responder (Mann et al., 2014; Hecksteden et al., 2015; Ahtiainen et al., 2016). Thus, one must expect that BSV will also occur in training responses in participants of experimental bed rest studies with countermeasures. Accordingly, the question arises how, statistically speaking, bed rest-related variability (*U*_*BR*_) interacts with countermeasure-related variability (*U*_*CM*_).

Hopkins (2015) as well as Atkinson and Batterham (2015) made a first statistical approach to quantify intervention-related BSV and compared it between the intervention group and the control group. Despite this existing approach and the possibility of linear mixed effect models, which provide an estimate of the overall variability by means of random factors, we would like to take a new statistical approach in this paper, which enables a differentiation between several components like *U*_*CM*_ and *U*_*BR*_ influencing the variability. Furthermore, we hypothesize that the combined bed rest- and countermeasure-related variability will be greater than after undergoing bed rest only. These results will show whether any performed countermeasure is an additional factor that needs to be taken into consideration while estimating the individuality of the musculoskeletal response. This can help to understand the variability within a crew for possible long-term missions, thereby estimating a possible health risk for a crew member who potentially has a higher loss of muscle and bone compared to the other crew members. Overall, this approach has an influence on future research enabling deep-space missions, as it is intended to give further insights into the extent to which variability in musculoskeletal response plays a role.

## 2 Methods

This work is based on data from already performed and published bed rest studies. We used this data for a novel statistical approach of data analysis to gain information to answer the specific research questions of this manuscript. All data was acquired after ethical approval by the ethics committee of the respective institution.

### 2.1 Selected studies, countermeasures and study design

In this secondary analysis, we included data of six bed rest studies conducted between 2001 and 2019 (Supplementary Material Table S1), which have been published in detail in Table 1 in Böcker et al. (2022). In addition to our previous paper (Böcker et al., 2022), we also included data from the experimental bed rest groups that received countermeasures. Starting chronologically, the long-term bed rest study (LTBR) as randomized controlled trial afforded two countermeasure groups beside one bed rest only group: one countermeasure group performed resistance training for knee extensors (3 sets of 7 repetitions) and for the foot plantar flexor muscles (3 sets of 14 repetitions) on a flywheel device on every third day (FW), with 2-3 weekly sessions each (Alkner and Tesch, 2004; Rittweger et al., 2005). The second LTBR countermeasure group received a single intravenous injection of pamidronate (PAM) prior to bed rest inhibiting bone resorption (Rittweger et al., 2005). The study was approved by the Ethical committee of the Rangueil University Hospital, Toulouse, France, before the inclusion of the first participant. During the Berlin Bed Rest (BBR) study (randomized control training study; ethical approval by Ethical Committee of the Campus Benjamin Franklin, Berlin, Germany), the countermeasure group performed resistive-vibration training (VbX) by using a vibration platform with added straps for resistive training (Rittweger et al., 2006). The Nutritional Countermeasure (NUC) study (randomized crossover design; ethical approval 2007405 by Ethical Committee of the Ärztekammer Nordrhein, Düsseldorf, Germany) provided a standardized dietary intervention to the countermeasure group that consisted in dietary supplementation of 90 mmol potassium per day (KHCO_3_) (Heer et al., 2014). In the Medium-term whey protein (MEP) study (controlled and randomized cross-over design; ethical approval 2010426 by the Ethical Committee of the Ärztekammer Nordrhein, Düsseldorf, Germany), the countermeasure group (PROT) received whey protein (0.6 g of protein per kg body weight and day) and bicarbonate (90 mmol per day) as an energy-balanced dietary supplement (Bosutti et al., 2016). The Reactive Jumps in a Sledge Jump system as a countermeasure (RSL) study (randomized controlled training study; ethical approval 2014105 by Ärztekammer Nordrhein, Düsseldorf, Germany) used a gravity-independent jump sledge system as a countermeasure. The countermeasure group (JUMP) performed 48 jumping training sessions during the 60 days of bed rest (Kramer et al., 2017). Finally, during the Artificial Gravity Bed Rest with ESA (AGBRESA) study (randomized controlled training study; ethical approval 2018143 by Ärztekammer Nordrhein, Düsseldorf, Germany), two countermeasure groups were observed undergoing artificial gravity on a short-arm human centrifuge. One of the groups underwent intermitted centrifugation (6 × 5 min per day) (iAG), and the other group underwent continuous centrifugation (30 min per day) (cAG) (Frett et al., 2020). It is important to note that some of the interventions differ significantly from another. Nevertheless, we decided to compare these interventions because space travelers also use very different training methods or diets during their missions (Macias et al., 2005; Kozlovskaya et al., 2015; Scott et al., 2021). In addition, the same measurement methodology is available for all of these included studies (see Section 2.3).

**TABLE 1:** Observed variabilities *(U*_*Obs*_ *)* of percent change for each study, intervention group and measurement site. P-values show the results of the Levene-test after study-wise Bonferroni adjustment for comparing the variances between bed-rest only groups and countermeasure groups. The results showed no significant differences for *U*_*Obs*_ between countermeasure and control group. cAG: continuous artificial gravity. iAG: intermitted artificial gravity (Frett et al., 2020). VbX: Whole Body Vibration plus resistive training (Rittweger et al., 2006). FW: Resistive training on a flywheel. PAM: Pamidronate supplementation (Rittweger et al., 2005). PROT: Whey protein plus potassium bicarbonate supplement (Buehlmeier et al., 2014). KHCO3: Potassium bicarbonate supplement (Heer et al., 2014). JUMP: Reactive jumping on a horizontal sledge (Kramer et al., 2017).

Study	Intervention	MUSCLE_38		MUSCLE_66		TIBIA_04		TIBIA_38		TIBIA_66		TIBIA_98	
		*U*_*Obs*_	p-value	*U*_*Obs*_	p-value	*U*_*Obs*_	p-value	*U*_*Obs*_	p-value	*U*_*Obs*_	p-value	*U*_*Obs*_	p-value
AGBRESA	cAG	13.3	1.0	25.5	1.0	12.2	1.0	0.7	0.24	0.4	1.0	5.2	1.0
	iAG	20.6	1.0	20.4	1.0	1.2	1.0	0.4	1.0	0.2	1.0	8.6	1.0
	CTRL	18.8	-	20.0	-	0.7	-	0.2	-	0.3	-	2.4	-
BBR	VbX	-	-	34.0	1.0	0.9	0.24	1.1	1.0	0.4	0.08	-	-
	CTRL	-	-	23.6	-	4.3	-	1.2	-	0.2	-	-	-
LTBR	FW	-	-	8.0	1.0	5.5	1.0	-	-	0.3	1.0	-	-
	PAM	-	-	15.0	1.0	8.7	0.42	-	-	0.6	1.0	-	-
	CTRL	-	-	5.4	-	23.6	-	-	-	0.9	-	-	-
MEP	PROT	-	-	-	-	1.4	1.0	0.4	1.0	0.1	1.0	-	-
	CTRL	-	-	-	-	0.6	-	0.2	-	0.1	-	-	-
NUC	KHCO3	-	-	-	-	0.5	1.0	0.2	1.0	0.2	1.0	-	-
	CTRL	-	-	-	-	0.4	-	0.1	-	0.2	-	-	-
RSL	JUMP	18.2	1.0	21.0	1.0	2.7	1.0	0.3	1.0	0.5	1.0	1.6	1.0
	CTRL	9.3	-	11.3	-	2.8	-	1.0	-	0.3	-	2.3	-

### 2.2 Participants

For our analysis, we included datasets of 53 participants who belonged to the control group (CTRL), which underwent bed rest only. Furthermore, we analyzed the datasets of 70 participants, who underwent or performed countermeasures in addition to bed rest. All participants gave written consent to the specific study. During baseline data collection (BDC) and after re-ambulation all participants were measured by a peripheral quantitative computed tomography.

### 2.3 Peripheral quantitative computed tomography measurement and analysis

Peripheral quantitative computed tomography (pQCT) is a valid method to assess the bone mineral content (BMC) as well as the cross-sectional area (CSA) of the muscles as an indicator for muscle mass. As the averaged greatest bone loss occurred 14 days after re-ambulation, we used datasets of R+14 for the adaptations of BMC instead of R+1 as we did for muscle CSA. Furthermore, R+14 was used for analyzing the variability in bone response as this measurement date was scheduled in all included studies. We focused on the lower extremity, and thus we analyzed the results of TIBIA_04, TIBIA_38, TIBIA_66, and TIBIA_98, where the number indicates the relative position of the tibia from distal to proximal. The CSA of the muscles was analyzed at the diaphyseal sites (TIBIA_38, TIBIA_66). A detailed description of the analysis process has been explained in the methods sections “Peripheral Quantitative Computed Tomography Measurements” and “Image Analysis and Data Processing” in Böcker et al. (2022). Since the selected measurement sites were somewhat inconsistent across the studies, not all studies provided datasets for each measurement site. Furthermore, the pQCT device varied from study to study, which was considered during the analysis process by assessing the measurement uncertainty of the different devices (see Table 2 in Böcker et al. (2022)). These results of the measurement uncertainty were also used in the analysis of this manuscript.

**TABLE 2:** Observed variability (*U*_*Obs*_) and countermeasure-related variability (*U*_*CM*_) of the intervention groups separated by study and region. The table shows that *U*_*CM*_ only explains a small proportion of *U*_*Obs*_ and is even negative in some cases. Negative results are obtained by subtracting *U*_*CM*_ and *U*_*Meas*_ from *U*_*Obs*_. cAG: continuous artificial gravity. iAG: intermitted artificial gravity. VbX: Whole Body Vibration plus resistive training. FW: Resistive training on a flywheel. PAM: Pamidronate supplementation. PROT: Whey protein plus potassium bicarbonate supplement. KHCO3: Potassium bicarbonate supplement. JUMP: Reactive jumping on a horizontal sledge.

Study	Intervention	MUSCLE_38		MUSCLE_66		TIBIA_04		TIBIA_38		TIBIA_66		TIBIA_98	
		*U*_*Obs*_	*U*_*CM*_	*U*_*Obs*_	*U*_*CM*_	*U*_*Obs*_	*U*_*CM*_	*U*_*Obs*_	*U*_*CM*_	*U*_*Obs*_	*U*_*CM*_	*U*_*Obs*_	*U*_*CM*_
AGBRESA	cAG	13.3	−5.6	25.1	5.2	12.1	11.5	0.7	0.6	0.4	0.1	5.2	2.8
	iAG	20.6	1.7	20.4	0.5	1.2	0.4	0.4	0.2	0.2	−0.1	8.6	6.2
BBR	VbX	-	-	34.0	10.4	0.9	−3.4	1.1	−0.1	0.4	0.2	-	-
LTBR	FW	-	-	8.0	2.6	5.5	−18.1	-	-	0.3	−0.6	-	-
	PAM	-	-	15.0	9.6	8.7	−15.0	-	-	0.6	−0.3	-	-
MEP	PROT	-	-	-	-	1.4	0.8	0.4	0.2	0.1	0.0	-	-
NUC	KHCO3	-	-	-	-	0.6	0.2	0.2	0.1	0.2	0.0	-	-
RSL	JUMP	18.2	8.9	21.0	9.7	2.7	−0.1	0.3	−0.7	0.5	0.2	1.6	−0.7

### 2.4 Statistics

For all statistical computations, we used R in its version 4.3.2 (www.r-project.org) and RStudio in its version 2023.03.01 (Posit Software, Boston, United States). We used Levene’s test (R-function “leveneTest” of the car-package in its version 3.1–2) to compare the variances of the individual percent change *pc*_*i*_ (individual percent change from Pre to Post) of the countermeasure groups (CM) and control groups (CTRL). A study-wise Bonferroni adjustment for multiple testing was carried out so that the p-values were multiplied by the corresponding factor depending on the number of tests within the respective study. Calculation of Cohen´s d enabled us to make a statement about the effect size of the countermeasures (R-function “cohen.d” of the effsize-package in its version 0.8.1).

This work extends the previous analysis in Böcker et al. (2022) and uses for example, the measurement uncertainty *U*_*Meas*_. As we focus in this work on differentiating between variation from bed rest and from countermeasure, we introduce the variables *U*_*BR*_ and *U*_*CM*_, respectively, and *U*_*Obs*_ as the combined observed uncertainty. *U*_*Obs*_ was defined in our previous work as the variance of the individual percentual loss *pc*_*i*_ of BMC or CSA after bed rest without a countermeasure. It is prudent to assume that countermeasure effectiveness η will affect *U*_*BR*_ although the exact nature of this effect is undetermined. Taking these considerations together, we propose the following equations:

$$U_{Obs}=U_{Meas}+f\left(\eta \right)\times U_{BR}+U_{BR}+U_{CM}$$ (1)

$$U_{CM}={U_{Obs}-U}_{Meas}-f\left(\eta \right)\times U_{BR}-U_{BR}$$ (2)

with *U*_*Obs*_ as the overall observed variability, *U*_*Meas*_ as the measurement uncertainty based on the pQCT device, *U*_*BR*_ as the variability of the individual response after bed rest only, *η* as the effectiveness of a countermeasure and *f(η)* as a function of the interaction of effectiveness of the countermeasure on *U*_*BR*_, respectively.

If we then divide Equation 2 by *U*_*BR*_, assuming that *U*_*BR*_ is not equal to 0, the result is

$$U_{OM}=\frac{U_{CM}}{U_{BR}}+f\left(\eta \right)+1$$ (3)

The crucial question then arises how function *f* is best modeled. For each countermeasure and measurement site, we calculated η as:

$$\eta =100\%\times \left(1-\frac{\bar{{pc}_{CM}}}{\bar{{pc}_{BR}}}\right)$$ (4)

with *pc*_*BR*_ as mean percent change after bedrest without countermeasure and *pc*_*CM*_ as mean percent change after bedrest with undergoing countermeasure.

For simplification of the problem, we define *U*_*OM*_ as uncertainty normalized to *U*_*BR*_ as

$$U_{OM}=\frac{U_{Obs}-U_{Meas}}{U_{BR}}$$ (5)

Equation 5 can therefore be used to statistically model function *f* that relate *U*_*OM*_ to η with offset *U*_*CM*_/*U*_*BR*_ *+1*.

To detect linear relationships between the effectiveness η and *U*_*OM*_, we used a linear regression model using the lm-function of R. Due to the present intra-individual variability as shown in our previous work (see Figure 5 in Böcker et al. (2022)), we differentiated between the epiphyseal and diaphyseal sites as well as muscle sites.

Furthermore, for comparing *U*_*Obs*_ of muscle and bone, we used the Shapiro test (R-function shapiro.test) for testing whether the data were normal distributed. Based on these results, we performed a t-test (R-function t. test) or a Wilcoxon test (R-function wilcox.test).

As additional analysis the approach of Hopkins (2015), who defined *SD*_*IR*_ as the standard deviation of the individual response as

$${SD}_{IR}=\sqrt{{{SD}_{\mathrm{Exp}}}^{2}-{{SD}_{\mathrm{Con}}}^{2}}$$ (6)

with *SD*_*Exp*_ and *SD*_*Con*_ as the standard deviation of the change score (absolute difference between pre- and post-intervention) of experimental and control group was performed. Atkinson and Batterham (2015) stated that in case of small *SD*_*IR*_ the inter-individual response to an intervention is negligible, but there is no definition for a small effect as it must set in relation of the adjustments, which occur.

## 3 Results

**FIGURE 1:**
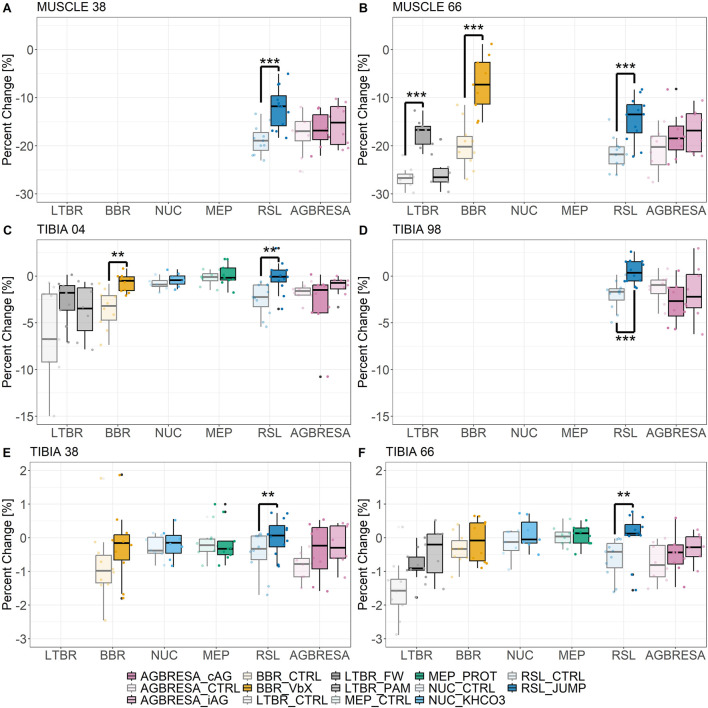
Muscle wasting and bone loss after experimental bed rest and countermeasure as boxplots for **(A)** MUSCLE_38, **(B)** MUSCLE_66, epiphyseal sites: **(C)** TIBIA_04, **(D)** TIBIA_98, diaphyseal sites: **(E)** TIBIA_38, and **(F)** TIBIA_66. ^*^ <0.05; ^**^ <0.01; ^***^ <0.001 indicates significant differences between countermeasure (CM) and bed rest only group (CTRL). In case of LTBR, the significant difference was between CTRL and FW and in case of AGBRESA between CTRL and iAG, respectively. Light coloring indicates the CTRL groups, bright coloring indicates the countermeasure groups. cAG: continuous artificial gravity. iAG: intermitted artificial gravity. VbX: Whole Body Vibration plus resistive training. FW: Resistive training on a flywheel. PAM: Pamidronate supplementation. PROT: Whey protein plus potassium bicarbonate supplement. KHCO3: Potassium bicarbonate supplement. JUMP: Reactive jumping on a horizontal sledge.

The percent changes of BMC and muscle CSA (Figure 1) revealed significant differences between some countermeasures and the control groups (Supplementary Material Table S2). In the RSL study, JUMP showed statistically significant protective effects at all measurement sites (all p ≤ 0.04; Cohen´s d all ≥0.915). Furthermore, there were effects for VbX for MUSCLE_66 (p < 0.001; Cohen´s d = 2.37) and TIBIA_04 (p < 0.01; Cohen´s d = 1.73) during BBR as well as FW for MUSCLE_66 (p < 0.001; Cohen´s d = 1.25) during LTBR. The remaining measurement sites of the several studies did not show any further significant effects of the countermeasures.

**FIGURE 2:**
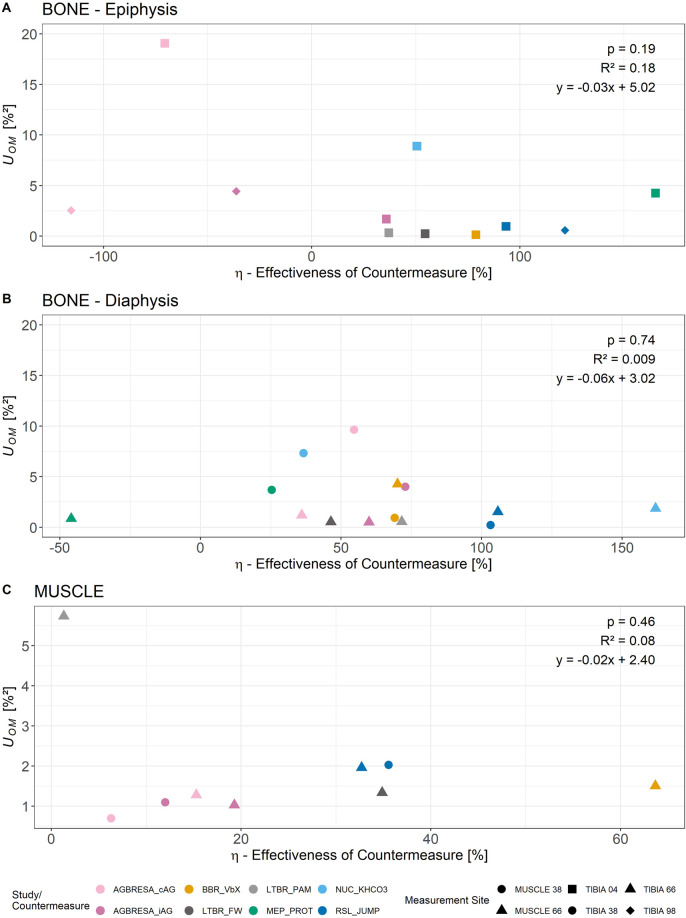
Relationship of η (effectiveness of countermeasure) and *U*_*OM*_ separated by measurement site and study/countermeasure. As in our previous work described (Böcker et al., 2022), there are intra-individual variations between measurement sites. Thus, the analysis was divided into epiphyseal bone sites **(A)**, diaphyseal bone sites **(B)**, and muscle sites **(C)**, respectively. The linear regression analysis revealed no statistically significant linear relationship between the two parameters.

Furthermore, observed variances (*U*_*Obs*_) showed no significant differences between intervention and control groups (Levene´s test with Bonferroni adjustment, all p ≥ 0.08). Fitting $f\left(\eta \right)$ from Equation 3 for epiphyseal bone sites (Figure 2A), diaphyseal bone sites (Figure 2B), and muscle sites (Figure 2C) yielded insignificant results (all p ≥ 0.19). Accordingly, we set $f\left(\eta \right)\times U_{BR}$ to 0 in Equation 2 and obtained:

$$U_{CM}={U_{Obs}-U}_{Meas}-U_{BR}$$ (7)

Both the magnitude of the losses (Figure 1), as well as *U*_*Obs*_ were greater for muscle measurement sites than for bone sites (p < 0.001, Table 1). More specifically, Wilcoxon’s test (*U*_*Obs*_ for bone sites not normally distributed, p < 0.001) revealed that muscle-*U*_*Obs*_ (median and Interquartile Range (IQR): 20.4 [6.0]) was greater compared to bone-*U*_*Obs*_ (0.6 [1.2], p < 0.001), and that muscle-*U*_*CM*_ was greater (5.2 [7.9]) than bone-*U*_*CM*_ (0.1 [0.5]) (p = 0.003).

Generally, greater loss magnitudes were associated with greater *U*_*Obs*_ and *U*_*CM*_. Thus, when comparing the epiphyseal (TIBIA_04, TIBIA_98) and diaphyseal bone sites, it was found that *U*_*Obs*_ was 10.8 times greater at epiphyseal sites than at diaphyseal sites (p < 0.001), whilst *U*_*CM*_ showed no differences between these groups (p = 0.77) (Table 2).

The analysis based on Hopkins (2015) showed a maximum value of 15.45 for SD_IR_ for iAG at TIBIA_98. In general, the values showed a range of 34.52 with the minimum being −19.12 (FW, TIBIA_04). Negative values were obtained due to the computation proposed by Hopkins, which converts negative values within the square root to negative square roots of the absolute of these negative values. In total, 19 out of 34 (55.9%) values were negative indicating negligible impact of a countermeasure in contrast to bed rest only, which in some cases had a greater impact than the performed countermeasures (Table 3).

**TABLE 3:** Results of calculation of standard deviation for the individual response SD_IR_ based on Hopkins (2015). Small and negative values indicate a negligible effect of countermeasure intervention on the overall inter-individual variation. According to the literature, there is no definition of when an effect is small (Atkinson and Batterham, 2015). The effect should be seen related to the adjustments that occur. Therefore, here the assumption was made that a value below 5 is small. cAG: continuous artificial gravity. iAG: intermitted artificial gravity. VbX: Whole Body Vibration plus resistive training. FW: Resistive training on a flywheel. PAM: Pamidronate supplementation. PROT: Whey protein plus potassium bicarbonate supplement. KHCO3: Potassium bicarbonate supplement. JUMP: Reactive jumping on a horizontal sledge.

Study	Intervention	MUSCLE_38	MUSCLE_66	TIBIA_04	TIBIA_38	TIBIA_66	TIBIA_98
		SD_IR_	SD_IR_	SD_IR_	SD_IR_	SD_IR_	SD_IR_
AGBRESA	cAG	2.97	4.20	12.04	1.21	−1.97	9.15
	iAG	1.40	−1.44	−2.30	−0.84	−2.19	15.45
BBR	VbX	-	−2.29	−7.92	1.87	−1.47	-
LTBR	FW	-	−1.86	−19.12	-	−2.47	-
	PAM	-	3.18	−16.96	-	−1.57	-
MEP	PROT	-	-	2.65	−0.40	0.25	-
NUC	KHCO3	-	-	−1.40	0.67	0.23	-
RSL	JUMP	1.10	4.07	−3.44	−3.96	−1.39	−11.92

## 4 Discussion

The aim of this study was to disentangle the sources of variability in the musculoskeletal response. It was anticipated that variability would be increased by countermeasures during bed rest compared to bed rest only. In contrast to that hypothesis, we rather found that the variability is not increased by the countermeasures and their influence on the total variability is negligible.

### 4.1 General losses and comparison of *U*_*Obs*_ between CM and CTRL groups

The study-specific results of the pQCT measurements have mostly appeared in publications dedicated to the specific studies. In our research, we combined all those studies and investigated the influence of countermeasures on subject group variability, which is new, using the largest data set in this area to date. The comparison of *U*_*Obs*_ between CM and CTRL showed no differences between the groups (Table 1). This indicates that the variability in the results of the individual study participants was just due to the effects of bed rest. A possible explanation is that the change from everyday life to bed rest outweighed the fact that there was a wide range in musculoskeletal response in relation to a countermeasure. This resulted in significantly greater variability due to bed rest (*U*_*BR*_). In contrast, the variability of being (non-) responder (*U*_*CM*_) was significantly smaller and therefore had no effect on the overall variability *U*_*Obs*_. In order to further understand the causes of variability, future studies may focus on bio (markers) that have been shown to change due to bed rest (Fernandez-Gonzalo et al., 2021). This should also be done on a sex-specific basis, as according to Ploutz-Snyder et al. (2014), the variability is also caused by gender differences, which were not considered in this study. However, it may still be too early to fully acknowledge sex effects on the response to immobilization, as women are largely under-represented in bed rest studies (in this data set 8 out of 123 participants).

### 4.2 U_*CM*_ in relation to *U*_*Obs*_

As described, there were no significant differences for *U*_*Obs*_ between CM and CTRL. This was also reflected in the results for *U*_*CM*_, which were small relative to *U*_*Obs*_. It has been shown that both *U*_*Obs*_ and *U*_*CM*_ were greater for muscle than for bone. If a distinction was made within the tibia, it could be seen that *U*_*Obs*_ differed between the epiphysis and diaphysis, but there were no differences for *U*_*CM*_. The decisive factor here was that the measurement uncertainty *U*_*Meas*_ was greater for TIBIA_04 and TIBIA_98 compared to TIBIA_38 and TIBIA_66, and the Uncertainty of Individual Response *U*_*IR*_ (referred to as *U*_*BR*_ in this paper) was greater in the epiphyseal measurement regions (see Table 4 in Böcker et al. (2022)). Our approach includes a possible interaction between the effectiveness of a countermeasure f(η) and the variability due to bed rest *U*_*BR*_. However, the results of the linear regression analysis showed no significant correlation for all measurement sites. Accordingly, the interaction term describing the relation of η to *U*_*OM*_ of Equation 2 was 0. This suggests that countermeasures have no sizable effects on *U*_*BR*_, even in the case of full countermeasure effectiveness, and that *U*_*CM*_ plays a subordinate role for overall *U*_*Obs*_. Transferring these results from bed rest to space would imply that the changed gravitational conditions have a major influence on BSV, but countermeasures may not further increase it.

**TABLE 4:** Calculation of potential bone loss at epiphyseal bone sites of the strongest responder of a crew of six (Bone loss of Crew #6). 0% represents a countermeasure with no effect, 100% a countermeasure which maintains the status before microgravity exposure. A mission duration of 12 months was assumed for this calculation. Mean bone loss of 1% per month with 0% countermeasure effectiveness was assumed, because the last two bed rest studies (AGBRESA, RSL) showed a monthly bone loss of approximately 1% for the control groups at the epiphyseal sites (Böcker et al., 2022).

Countermeasure effectiveness (η)	Mean bone loss per month	Mean bone loss	Additional bone loss of crew #6	Bone loss of crew #6
0%	1%	12%	11.6%	23.6%
25%	0.75%	9%	8.7%	17.7%
50%	0.5%	6%	5.8%	11.8%
75%	0.25%	3%	2.9%	5.9%
100%	0%	0%	0%	0%

### 4.3 Comparison of *U*_*CM*_ for several countermeasures

A comparison of the different countermeasures revealed differences in *U*_*CM*_ for the muscle regions examined. *U*_*CM*_ was higher after intensive training with high impacts (JUMP) and resistance training with vibration (VbX), and lower for endurance training (FW) and passive countermeasures (cAG, iAG) (Table 2). From literature, higher-load resistance exercises and high-intensity interval training as provided by JUMP and VbX could minimize microgravity induced muscle and bone adaptations (Kramer et al., 2017; Scott et al., 2021; Rittweger et al., 2010) compared to passive countermeasures as centrifugation (Smith et al., 2009). There was no difference in outcome for astronauts exercising with high intensity compared to standard exercising on ISS, thus, crew time was saved, which could be used for other tasks (English et al., 2020).

In addition to the very different countermeasures, which had a wide range of training stimuli, there were other factors that were responsible for the differences in *U*_*CM*_. Compared to the other studies, AGBRESA was the only study where both males and females were included (Frett et al., 2020). Furthermore, the participants in AGBRESA, RSL, BBR and LTBR differed in body mass index (BMI) (Frett et al., 2020; Kramer et al., 2017; Rittweger et al., 2006; Rittweger et al., 2005), which was lower for AGBRESA and LTBR. If PAM is neglected, a tendency can be recognized that *U*_*CM*_ was smaller for the groups with the smaller BMI (cAG, iAG, FW). However, due to the present data, this could not be statistically verified. These tendencies could not be obtained for the bone sites at all. Furthermore, there are many other factors like diet, lifestyle habits, and health status that have an influence on *U*_*CM*_ (Liphardt et al., 2023), but could not be quantified further due to the data used.

### 4.4 Statistical approach compared to individual response by hopkins

To test this novel approach, the standard deviation of the individual response *SD*_*IR*_ was also calculated according to Hopkins (2015) and Atkinson and Batterham (2015). According to Scott et al. (2021), it is generally the case in bed rest studies that people who do not experience countermeasures are regarded as a control group (Scott et al., 2021). The established approach, which is based on the change score (absolute difference between pre- and post), supports the approach developed in this paper. 55.9% of the calculated *SD*_*IR*_ are negative, another 34.3% are below 5. According to the literature, there is no definition of when an effect is small (Atkinson and Batterham, 2015). The effect should be seen related to the adjustments that occur. Therefore, the assumption was made here that a value below 5 is small. The effects of the countermeasures were, therefore, negligible compared to the influence of bed rest. In our opinion, our approach has the advantage over Hopkins (2015) that it takes the measurement uncertainty into account as Atkinson and Batterham stated that small *SD*_*IR*_ are based on within-subject variation and measurement noise (Atkinson and Batterham, 2015). This can have a major influence on the interpretation of the results, especially for measurements where only very small changes are expected.

In total, it can therefore be concluded that the individual response to a countermeasure is negligible in comparison to the individual response to bed rest. Thus, in the context of future long-term missions, the individual adaptations to microgravity play a greater role as the variability in effectiveness of a countermeasure.

### 4.5 Calculation of individual health risks of the crew members

Results of this study can now be used, e.g., by crew surgeons, for predicting crew risks for future long-term missions. As previously suggested, one would predict a 23.6% loss in epiphyseal tibia bone mass after 6 months of weightlessness in the strongest responder in a crew of six (Böcker et al., 2022). If the maximum bone loss is now to be calculated for a crew of six that carries out regular countermeasures, this depends largely on the effectiveness of these countermeasures. Based on the calculation in the discussion section “Preventing Worst Case Scenarios” by Böcker et al. (2022) the equation is as follows:

$${BL}_{\#6}=MD\times {BL}_{mean/month}+0.967\times MD\times {BL}_{mean/month}$$ (8)

with *BL*_*#6*_ as the calculated bone loss of the strongest responder, *MD* as mission duration, *BL*_*mean/month*_ as the mean bone loss per month, and 0.967 as the upper tail quantile for 1/6 of the normal distribution, respectively.

For example, a mission duration of 12 months and a countermeasure effectiveness of 50% leads to a maximum bone loss of 11.8%. An individual bone loss of up to 23% and well about 12% has already been published, so a countermeasure effectiveness of about 50% would ensure the success of a deep-space, long-lasting space mission (Sibonga et al., 2015; Sibonga et al., 2007; Vico et al., 2000).

### 4.6 Limitations and strengths

This study has some limitations, which, however, do not affect the overall conclusions. The main limitation is the difference in interventions, which ranged from supplements to training interventions. But space travelers undergo several training and diet regimens with large differences (Scott et al., 2021; Macias et al., 2005; Kozlovskaya et al., 2015), thus, we decided to include all data sets. Furthermore, due to the different study designs, results were not available for every measurement region in all studies. In this case, however, it was more important for us to include a data set as large as possible in the analysis so that we could make more general statements. To our knowledge, this study includes the largest and most comprehensive dataset to date investigating multiple interventions during bed rest, allowing a detailed analysis of musculoskeletal variability. We defined R+14 for analyzing the variability of bone response as previous studies showed that greatest average bone loss occurred at this time point. This means that the temporal component of the variability cannot be recorded, as there may be inter-individual differences in when the maximum bone loss occurs. However, our approach enabled us to generate the largest possible data set, because corresponding measurements were carried out on R+14 in all included studies. In this study, we also did not explore the effects of sex. That omission is due to the historical reluctance to include women in bed rest studies, as in this case, only AGBRESA enabled women´s participation. Furthermore, the studies included in our data set had differing protocols, ranging from 21 to 90 days of bed rest, and included different kinds of countermeasures, but as stated before, it was the aim of this work to get the largest data set possible. Possibly, because of the differences in countermeasures no linear relationship of *U*_*OM*_ and η was obtained, but the study focused on the inclusion of as many different countermeasures as possible to get a more general statistical approach. The fact that the decrease in muscle and bone mass is not linear, as shown in previous publications, may, admittedly, affect the numerical values of *U*_*Obs*_ and *U*_*CM*_. However, we feel that this will be unlikely to introduce bias into the within-study comparisons with the control group. Finally, the duration of all included studies was significantly shorter compared to deep space missions. However, it is ethically difficult to justify extending the duration of bed rest even further and previous analysis showed that about 60 days of bed rest elicits similar musculoskeletal adaptations as after 6 months of microgravity exposure (Hargens and Vico, 2016). It must, therefore, be taken into account that *U*_*CM*_ increases with increasing duration and could possibly still have an influence on the BSV contrasting the results of this work. However, this is the first study to show that a countermeasure during a bed rest study does not increase the variability and thus the inter-individual variability. The aim is to make a further contribution to better understanding adaptations to the changed environmental conditions in order to be able to develop appropriate countermeasures on this basis (Konda et al., 2019).

## 5 Conclusion

The aim of this work was to show that the variability increases as soon as a person undergoes a countermeasure to reduce muscle and bone loss in addition to bed rest. However, it was shown that the variability induced by bed rest had a significantly greater influence on the observed variability and that the influence of the countermeasures was negligible. Furthermore, countermeasures did not reduce the variability caused by bed rest. It, therefore, plays a subordinate role whether a crew member is rather a responder or non-responder to a training intervention, but rather how the individual reacts to the changed environmental conditions. This fact can be used during future crew selection, especially for long-duration deep-space missions. Additionally, our approach included a possible interaction of countermeasure and bed rest, but our analysis did not obtain any linear relationship. For future approximations of possible bone and muscle loss during long-term missions, the effect of microgravity and countermeasures on the musculoskeletal system can be calculated separately with a primary focus on the effect of microgravity. Future research should investigate whether targeted measures, such as activation of satellite cells, prior to immobilization or weightlessness can influence the effect of countermeasures positively. Furthermore, during future bed rest campaigns it should be determined which factors lead to larger bone loss and muscle wasting increasing the variability between participants.

## Data Availability

The data analyzed in this study is subject to the following licenses/restrictions: Some of the data was generated as part of ESA studies and this data can be requested directly from ESA. Requests to access these datasets should be directed to JB, jonas.boecker@dlr.de.
