# Supplementary material for: Variability in individual musculoskeletal response is not increased by countermeasures during bed rest
Source: Front Physiol. 2025 Oct 14;16:1645482. doi: 10.3389/fphys.2025.1645482 (PMC12558760; doi:10.3389/fphys.2025.1645482)
Supplement: Supplementary file 1 [file DataSheet1.pdf]

Supplementary Material

Supplementary Table 1: Overview about the included studies with information about the year, the number of subjects and the intervention. HDT: head-down tilt bed rest (6°). HBR: horizontal bed rest. cAG: continuous artificial gravity. iAG: intermitted artificial gravity. VbX: Whole Body Vibration plus resistive training. FW: Resistive training on a flywheel. PAM: Pamidronate supplementation. PROT: Whey protein plus potassium bicarbonate supplement. KHCO3: Potassium bicarbonate supplement. JUMP: Reactive jumping on a horizontal sledge.

Study	Year	Number of Subjects	Bed Rest Duration	Intervention	Conclusions
AGBRESA (Frett et al., 2020)	2019	8	60	CTRL: HDT	Countermeasures not effective against loss of bone mineral content and maximum voluntary contraction of the knee extensors (Scott et al., 2021)
		8		cAG: continuous artificial gravity via centrifugation for 30 minutes per day	
		8		iAG: intermittent artificial gravity via centrifugation for 6x5 minutes per day	
BBR (Rittweger et al., 2006)	2003/2004	10	56	CTRL: HBR	Resistive vibration exercise maintained bone mass, but could not completely mitigate muscle atrophy (Rittweger et al., 2010)
		10		VbX: 89 exercise sessions per subject with combination of resistive exercises and vibration	
LTBR (Alkner and Tesch, 2004, Rittweger et al., 2005)	2001/2002	9	90	CTRL: HDT	Flywheel resistance training had the potential to prevent muscle atrophy and both countermeasures may be beneficial to preserve bone mineral content (Rittweger et al., 2005)
		9		FW: Flywheel resistance training, where resistance was provided during concentric and eccentric actions	
		7		PAM: 60mg intravenous pamidronate 14 days prior to bed rest	
MEP (Bosutti et al., 2016)	2011/2012	8	21	CTRL: HDT	There were no differences between CTRL and PROT in loss of muscle cross sectional area of the calf (Bosutti et al., 2020)
		9		PROT: supplementation of 0.6 g whey protein/kg body weight/day and 90 mmol of potassium bicarbonate ($KHCO_3$)/day	
NUC (Heer et al., 2014)	2010	7	21	CTRL: HBR	There were no differences between CTRL and $KHCO_3$ for bone mineral content loss (see Figure 1 of this article)
		7		$KHCO_3$: 90 mmol potassium bicarbonate ($KHCO_3$)/day	
RSL (Kramer et al., 2017)	2015/2016	11	60	CTRL: HDT	Plyometric exercises were very effective and maintained bone mineral content and maximum voluntary contraction of the knee extensors (Scott et al., 2021)
		12		JUMP: 48 training sessions consisting of plyometric exercises	

Supplementary Table 2: Results of the comparison of percent changes between control and intervention expressed by p-values. Significant differences are marked in grey. As for AGBRESA and LTBR multiple statistical tests were performed, a Bonferroni adjustment was performed. cAG: continuous artificial gravity. iAG: intermitted artificial gravity. VbX: Whole Body Vibration plus resistive training. FW: Resistive training on a flywheel. PAM: Pamidronate supplementation. PROT: Whey protein plus potassium bicarbonate supplement. KHCO3: Potassium bicarbonate supplement. JUMP: Reactive jumping on a horizontal sledge.

Study	Intervention	MUSCLE_38	MUSCLE_66	TIBIA_04	TIBIA_38	TIBIA_66	TIBIA_98
AGBRESA	cAG	1	0.40	0.72	0.38	0.74	0.30
	iAG	0.72	0.18	0.46	0.06	0.18	1
BBR	VbX	-	<0.001	<0.01	0.27	0.36	-
LTBR	FW	-	<0.001	0.18	-	0.12	-
	PAM	-	1	0.62	-	0.06	-
MEP	PROT	-	-	0.50	0.85	0.88	-
NUC	KHCO3	-	-	0.29	0.66	0.24	-
RSL	JUMP	<0.001	<0.001	<0.01	0.04	0.02	<0.001

References

ALKNER, B. A. & TESCH, P. A. 2004. Efficacy of a gravity-independent resistance exercise device as a countermeasure to muscle atrophy during 29-day bed rest. *Acta Physiol Scand.,* 181**,** 345-357.

BOSUTTI, A., MULDER, E., ZANGE, J., BUHLMEIER, J., GANSE, B. & DEGENS, H. 2020. Effects of 21 days of bed rest and whey protein supplementation on plantar flexor muscle fatigue resistance during repeated shortening contractions. *Eur J Appl Physiol,* 120**,** 969-983.

BOSUTTI, A., SALANOVA, M., BLOTTNER, D., BUEHLMEIER, J., MULDER, E., RITTWEGER, J., YAP, M. H., GANSE, B. & DEGENS, H. 2016. Whey protein with potassium bicarbonate supplement attenuates the reduction in muscle oxidative capacity during 19 days of bed rest. *J Appl Physiol (1985),* 121**,** 838-848.

FRETT, T., GREEN, D. A., MULDER, E., NOPPE, A., ARZ, M., PUSTOWALOW, W., PETRAT, G., TEGTBUR, U. & JORDAN, J. 2020. Tolerability of daily intermittent or continuous short-arm centrifugation during 60-day 6o head down bed rest (AGBRESA study). *PLoS One,* 15**,** e0239228.

HEER, M., BAECKER, N., WNENDT, S., FISCHER, A., BIOLO, G. & FRINGS-MEUTHEN, P. 2014. How Fast Is Recovery of Impaired Glucose Tolerance after 21-Day Bed Rest (NUC Study) in Healthy Adults? *The Scientific World Journal,* 2014**,** 803083.

KRAMER, A., KÜMMEL, J., MULDER, E., GOLLHOFER, A., FRINGS-MEUTHEN, P. & GRUBER, M. 2017. High-Intensity Jump Training Is Tolerated during 60 Days of Bed Rest and Is Very Effective in Preserving Leg Power and Lean Body Mass: An Overview of the Cologne RSL Study. *PLoS One,* 12**,** e0169793.

RITTWEGER, J., BELAVY, D., HUNEK, P., GAST, U., BOERST, H., FEILCKE, B., ARMBRECHT, G., MULDER, E., SCHUBERT, H., RICHARDSON, C., DE HAAN, A., STEGEMAN, D. F., SCHIESSL, H. & FELSENBERG, D. 2006. Highly demanding resistive vibration exercise program is tolerated during 56 days of strict bed-rest. *Int.J Sports Med,* 27**,** 553-559.

RITTWEGER, J., BELLER, G., ARMBRECHT, G., MULDER, E., BUEHRING, B., GAST, U., DIMEO, F., SCHUBERT, H., DE, H. A., STEGEMAN, D. F., SCHIESSL, H. & FELSENBERG, D. 2010. Prevention of bone loss during 56 days of strict bed rest by side-alternating resistive vibration exercise. *Bone,* 46**,** 137-147.

RITTWEGER, J., FROST, H. M., SCHIESSL, H., OHSHIMA, H., ALKNER, B., TESCH, P. & FELSENBERG, D. 2005. Muscle atrophy and bone loss after 90 days' bed rest and the effects of flywheel resistive exercise and pamidronate: results from the LTBR study. *Bone,* 36**,** 1019-29.

SCOTT, J. P. R., KRAMER, A., PETERSEN, N. & GREEN, D. A. 2021. The Role of Long-Term Head-Down Bed Rest in Understanding Inter-Individual Variation in Response to the Spaceflight Environment: A Perspective Review. *Front Physiol,* 12**,** 614619.
